# A Case Report of Disseminated Intravascular Coagulation After Intrauterine Fetal Demise

**DOI:** 10.7759/cureus.88728

**Published:** 2025-07-25

**Authors:** Gabrielle A Agcaoili, Kyriaki V Schooley, Elizabeth Johnson-Gray, Vincent Relli-Dempsey, Greeshma Allareddy

**Affiliations:** 1 Obstetrics and Gynecology, University of Arkansas for Medical Sciences, Little Rock, USA; 2 Anesthesiology, Ohio University Heritage College of Osteopathic Medicine, Dublin, USA; 3 Anesthesiology, OhioHealth Doctors Hospital, Columbus, USA

**Keywords:** coagulopathy, dic, iufd, obstetrics & gynecology, pregnancy

## Abstract

Disseminated intravascular coagulation (DIC) is a rare, life-threatening complication of pregnancy in which a hypercoagulable state develops secondary to the contact of tissue factor with circulating blood and causes widespread abnormal clotting. This pathological process disrupts normal blood flow, which can cause multi-organ dysfunction and is associated with very high maternal morbidity and mortality. Occurring in two phases, DIC begins with overactive clotting, which leads to the formation of blood clots within the vessels, and ultimately, the overactive clotting process uses up the body’s stores of platelets and clotting factors. The most common obstetric cause of DIC is placental abruption, but other common causes include the hemolysis, elevated liver enzymes, low platelet count (HELLP) syndrome and amniotic fluid embolism. The management of DIC involves the administration of antibiotics in the setting of severe sepsis, emergent delivery in the case of placental abruption, and transfusions of packed red blood cells (PRBCs), platelets, and plasma as needed for patients with active bleeding and unstable labs. As evidenced in this case, timely recognition and treatment of the underlying cause of DIC is imperative in improving patient outcomes. Here, we present a case of a 37-year-old G3P2 32w3d female who experienced placental abruption, resulting in intrauterine fetal demise, subsequently leading to DIC.

## Introduction

Disseminated intravascular coagulation (DIC) is a hypercoagulable state wherein widespread abnormal clotting can disrupt normal blood flow and clotting, leading to multiorgan dysfunction and systemic complications [1-3]. In pregnancy, DIC can occur in emergency settings, such as placental abruption and amniotic fluid embolism, as well as in more commonly seen conditions such as pre-eclampsia [4,5]. DIC typically develops in two distinct phases. Initially, widespread clot formation occurs within the blood vessels. As this process continues, the body rapidly consumes platelets and clotting proteins, leaving it unable to form clots when needed and increasing the risk of bleeding. Outward symptoms of this condition include bleeding from multiple sites of the body, bruising, and blood present in the urine or stool [2,3]. In rare cases, DIC can lead to death for both the mother and infant [6].

Premature separation of the placenta, or abruptio placentae, is the most frequent obstetric cause of coagulation failure [1]. The procoagulant environment of the placenta raises the likelihood of clotting complications during pregnancy. When activated locally, these clotting processes can extend into the circulatory system, leading to widespread involvement. A DIC score is helpful in diagnosis and treatment, and has three components: 1) the underlying diseases, 2) the clinical symptoms, and 3) the laboratory findings (coagulation tests) [7]. Clinical management typically recommends starting treatment for DIC when the obstetric DIC score reaches 8 or higher, even before laboratory results are available [8,9]. Supportive treatment for DIC is aimed at replacing platelets and coagulation factors, providing anticoagulant treatment, and restoring normal anticoagulant pathways as well as emergent removal of the placenta [5,7].

Early recognition and management of DIC are critical, and this case report presents a patient determined to have DIC following placental abruption and intrauterine fetal demise at 32 weeks of gestation. This case report demonstrates the treatment of DIC upon identification.

## Case presentation

A 37-year-old G3P2 32w3d female with a past medical history of epilepsy, asthma, and depression presented via EMS to the emergency department for heavy vaginal bleeding and pain. She weighed 89 kilograms with a BMI of 32. Current medications included sertraline, lamotrigine 10mg BID, and folic acid. On arrival, the patient told providers that the last time she had felt fetal movement was nine hours prior. The obstetrics team was consulted, and on transabdominal ultrasound, she was found to have no fetal heart tones, and the transvaginal ultrasound was consistent with placental abruption. Admission labs demonstrated abnormalities in her hemoglobin and fibrinogen (Table 1). Her initial blood pressure was 132/72, and she was afebrile with a temperature of 37 degrees Celsius, saturating 98% on room air. She was admitted to the Labor and Delivery floor for observation and possible cesarean section. However, on arrival to the floor, she continued to bleed and started to become hypotensive with a systolic blood pressure reading of 90/40, with statements that she was feeling dizzy.

**Table 1:** Patient’s labs on admission

Parameters	Patient’s Lab Value on Admission	Reference Ranges
Hemoglobin	8.6 mg/dL	12-15 mg/dL
Platelets	205,000 K/mcL	150,000-450,000 K/mcL
Fibrinogen	28 mg/dL	200-400 mg/dL
International normalized ratio (INR)	3.1	0.8-1.1

At this time, anesthesia was called, and the decision was made to proceed with an emergent cesarean section to remove fetal tissue in the setting of active hemorrhage and concern for possible DIC. On arrival to the OR, the patient had a blood pressure reading of 125/86, a temperature of 37.1°C, and had an oxygen saturation of 97% on room air. She was induced with 150 mcg of propofol and 140 mcg of succinylcholine and was intubated with a 6.5 mm endotracheal tube. Initial end tidal CO2 was 28. After induction, another large-bore 18-gauge IV was placed along with an arterial line in her left radial artery, and a triple-lumen CVC was placed in her right internal jugular vein. Her blood pressure after induction was 95/67, and she responded to a bolus of 100 mcg of phenylephrine. At that time, repeat labs showed a drop in her hemoglobin to 6.9 and fibrinogen to 25, putting her at a very significant risk of bleeding. Per the obstetrician's note, after the delivery of the baby, there was an indentation on the maternal surface of the placenta and a developing retroplacental hematoma. Throughout the case, she received 3 units of packed red blood cells (PRBCs), 2 units of fresh frozen plasma (FFP), 2 units of platelets, 2.3 L of lactated Ringer's (LR), 1 unit of cryoprecipitate, 1 unit of tranexamic acid (TXA), intrauterine pitocin, and 1000 mcg of Cytotec per rectum. Her total quantitative blood loss was estimated at 3897 ml.

The patient remained intubated and was transferred to the ICU in critical condition in the setting of DIC secondary to hemorrhage, acute blood loss anemia, and hypovolemic shock. On arrival to the ICU, repeat labs showed a hemoglobin of 8.7, potassium of 7, and fibrinogen of 234. Out of concern for her critical potassium value, she was treated with Lokelma, calcium gluconate, insulin, and dextrose. Repeat labs at 24 hours showed potassium of 4.5, hemoglobin of 6.2, and fibrinogen of 261 (Table 2). 

**Table 2:** Patient’s labs on arrival to the ICU and 24 hours after admission to the ICU

Parameter	Labs on Arrival to the ICU	Labs 24 Hours After Admission to the ICU	Reference Ranges
Hemoglobin	8.2 mg/dL	6.7 mg/dL	12 - 15 mg/dL
Potassium	7 mEq/L	4.5 mEq/L	3.5 - 4.5 mEq/L
Fibrinogen	234 mg/dL	261 mg/dL	200 - 400 mg/dL
Platelets	141,000 K/mcL	222,000 K/mcL	150,000-450,000 K/mcL
International normalized ratio (INR)	1.2	1.1	0.8-1.1
Creatinine	0.4 mg/dL	1.8 mg/dL	0.4-1.1 mg/dL

Although her fibrinogen and potassium values had normalized at this point in her admission, she required two additional units of PRBCs, was extubated on post op day (POD) 1, and transferred out of the ICU on POD 2. Her hospital course was complicated by a mild acute kidney injury (AKI) and preeclampsia in the postpartum period on POD 2. Her initial creatinine on presentation to the hospital was 0.4, and her creatinine on day 2 was 1.8. The AKI resolved with IV fluids, and on discharge, her creatinine had returned to 0.4 (Table 2).

Along with the AKI development on POD 2, she developed a headache, BP readings showing systolic > 150 with her highest reading of 198/140, and a protein: creatinine ratio of 0.7. She was subsequently started on IV magnesium and oral Labetalol (200 mg) every 12 hours. Due to the amount of blood products received intra-operatively, she was also given a one-time dose of 20 mg IV lasix. On POD 4, her magnesium and labetalol were discontinued, and her BP had stabilized to 120-130s systolic. She was transitioned to PO Labetalol on POD 4. Blood pressure at that time was 115/86.

She was discharged on POD 6 and seen in the office two weeks later with no bleeding or passage of clots. Per chart review, she was also further counseled on her diagnosis of DIC, with explanations of the causes and likelihood of this event should she desire another pregnancy in the future. She was also encouraged to follow up with a mental health provider.

## Discussion

DIC is a complication associated with severe or life-threatening medical diagnoses. DIC is found to occur in 10% of all placental abruption cases, with the incidence of DIC in the setting of placental abruption being much higher in cases where fetal death has occurred [8]. DIC is defined as a consumptive coagulopathy caused by the contact of tissue factor with circulating blood [5]. Once tissue factors enter circulation, they can activate the clotting system by interacting with clotting proteins, ultimately converting prothrombin to thrombin. This cascade leads to the formation of widespread clots, which can severely disrupt normal blood flow throughout the body [10]. In DIC, the body may either break down clots too quickly or, in some cases, struggle to dissolve clots properly. Labs that are indicative of DIC include decreased levels of platelets, low fibrinogen (<150), and prolonged INR (>1.1) [2].

Placental abruption most often happens in the third trimester, though the exact cause isn’t always clear. Common risk factors include uterine trauma, a history of abruption, multiple pregnancies, high blood pressure, diabetes, preeclampsia, tobacco or drug use, older maternal age, fibroids, clotting disorders, early rupture of membranes, and uterine infections [11]. Placental abruption occurs in approximately 1% of pregnancies, and intrauterine fetal demise (IUFD) occurs in a reported 20% to 40% of pregnancies [8]. Hypofibrinogenemia, elevated levels of fibrinogen degradation products, and decreased clotting factor activity occur in 30% of cases of placental abruption with IUFD [1,8].

The treatment for DIC necessitates treating the underlying disorder, including antibiotics for severe sepsis, possible delivery for placental abruption, and consideration for surgical intervention in trauma-related cases [3,6]. Platelet and plasma transfusions should only be considered in patients with active bleeding or a high risk of bleeding or those requiring an invasive procedure. Fresh frozen plasma and cryoprecipitate can be transfused to replenish coagulation factors. Heparin may also be needed for extensive clotting. For patients with DIC who are not currently bleeding, preventive treatment with heparin or low molecular weight heparin is often recommended to reduce the risk of dangerous clotting [2]. As our patient presented in her third trimester with placental abruption, this put her at high risk for DIC. She was given six units of packed red blood cells, two units of fresh frozen plasma, and one unit of cryoprecipitate to restore coagulation factors, fibrinogen, and prothrombin time activity, demonstrating how vital this stabilization is in terms of improving long-term outcomes. Up to 25% of maternal deaths are attributed to DIC, making its recognition of utmost importance to intervene early and optimize patient outcomes [12].

## Conclusions

DIC in the setting of placental abruption and intrauterine fetal demise is a rare but life-threatening medical complication that requires prompt identification and treatment with intrapartum coagulation factors to facilitate delivery as well as correct hypofibrinogenemia in order to minimize maternal morbidity and mortality. As evidenced in this patient’s presentation, abnormal lab values, including an increased partial thromboplastin time and decreased fibrinogen level as hallmarks of the disease. Unfortunately, patients with DIC can quickly develop multi-system failure and experience high mortality rates, so recognition and rapid treatment of the cause of DIC itself is of utmost importance. Future considerations of patients who have had DIC should include optimizing the recovery period and the prevention and management of irreversible multi-organ failure. This may include advancements in earlier diagnosis and intervention, as well as personalized medicine that tailors intervention based on differing DIC etiologies.
